# ADDRESSING CURRICULUM DESIGN CHALLENGES: THREE DELIVERY DESIGN OPTIONS FOR UNDERGRADUATE AGING AND HEALTH COURSE

**DOI:** 10.1093/geroni/igad104.2111

**Published:** 2023-12-21

**Authors:** Mary Theresa Pagan, Minjung Seo

**Affiliations:** SUNY Oswego, Oswego, New York, United States; SUNY Oswego, Oswego, New York, United States

## Abstract

Providing a comprehensive gerontology course to diverse academic majors is a curriculum design challenge. Current research indicates students seek out the delivery option best suited to their needs, requiring educators to design or modify courses to provide face-to-face, hybrid, and online opportunities. Further, gerontologists are challenged with initial student knowledge and views on aging, aging processes, and health disparities shaped primarily by personal/life experiences with older adults and media representations of aging. Significant design and quality assurance were minimized by utilizing the recognized work of established gerontologists providing the overall framework for a comprehensive aging course Segmenting the course into two sections, social determinants of health utilizing Deborah Carr’s Golden Years? Social Inequalities in Later Life and target population research, Grandmothers at Work, Juggling Families and Jobs by Madonna Harrington Meyer. As a research design, a mixed method design (questionnaire and interview was used on seventy-eight students (Male: 27, Female: 51) enrolled in face-to-face, hybrid, and online delivery options. As a result of attending the three-credit course, significant knowledge-base increases were found in four key content areas: 1) social determinants of health (83%), 2) theories (sociological and biological)-(90%), 3) socioeconomic (SES) variations (80), and 4) mixed methods research design (62%). A curated library of online resources (youtube, Ted Talks, documentaries, news reports) supplemented the course textbooks for each learning module, resulting in a ninety-six percent resource satisfaction evaluation average for the three delivery methods.

